# Identification and management of unrecognized femoral head epiphysiolysis: a case report

**DOI:** 10.3389/fped.2026.1790075

**Published:** 2026-06-10

**Authors:** Martina Ranieri, Maria Rizzo, Francesco Maria Lotito, Alessandro Aprato, Maria Alessio, Valentina Discepolo, Marina Russo

**Affiliations:** 1 Department of Translational Medical Science (DISMET), Section of Pediatrics, University of Naples Federico II, Naples, Italy; 2 Department of Public Health, Orthopedic Unit, University of Naples Federico II, Naples, Italy; 3 Department of Surgical Sciences, University of Turin, Turin, Italy; 4 European Laboratory for the Investigation of Food Induced Diseases (ELFID), University of Naples Federico II, Naples, Italy

**Keywords:** case report, limping in children, obesity, SCFE, slipped capital femoral epiphysis (SCFE)

## Abstract

**Background:**

Slipped capital femoral epiphysis (SCFE) is the slippage of the proximal femoral epiphysis on the femoral neck through the epiphyseal plate. This condition is generally observed in male, obese patients during pubertal growth spurts, and it can sometimes be associated with endocrine disorders. Despite its significant incidence in the paediatric population, the diagnosis is frequently delayed which leads to significant worsening of the prognosis. We present a case of SCFE in which the diagnosis was delayed due to a series of confounding factors.

**Case presentation:**

We present a case of a 16-year-old boy who came to our rheumatology centre due to persistent limp for several months. Previous investigations aimed to exclude orthopaedic, infectious or inflammatory conditions, apparently revealed nothing significant, so he was referred for a rheumatological evaluation. Upon further investigation, a SCFE was identified and treated surgically with the modified Dunn procedure. Additionally, during the evaluation, endocrinological conditions such as insulin resistance and delayed puberty were discovered.

**Conclusion:**

This case shows that, even in the presence of a suggestive medical history and clinical picture, a common diagnosis such as SCFE can be delayed. The delay in this diagnosis is correlated with clinical worsening of the patient and the need for more invasive management. Therefore, SCFE should always be considered in an adolescent boy who starts experiencing a limp or pain in the lower limbs, even if poorly localized, especially because the diagnosis can be quickly made with a pelvic x-ray. Furthermore, it is always important to remember that conditions such as SCFE, can be associated with endocrinological comorbidities.

## Background

Slipped capital femoral epiphysis (SCFE) is defined as the displacement of the proximal femoral epiphysis relative to the metaphysis through the growth plate (physis). In the vast majority of cases, this displacement occurs in a posterior and inferior direction; however, atypical cases have been described in which the direction of slippage is posterior and lateral (valgus SCFE) (1–3). The aetiology of SCFE is multifactorial, but it is generally observed in male, obese patients (>50% of the patients are above the 95th percentile for weight) during pubertal growth spurts, and it can be associated with endocrine disorders such as hypothyroidism, hypopituitarism, hypogonadism or during growth hormone replacement therapy (1).

The incidence is between 0.33 and 24.58 cases per 100.000 children between 8 and 15 years of age, with a mean age of 12.0 years for boys and 11.2 years for girls. An increased risk of SCFE has been reported among African American, Polynesian, Indigenous Australian, and Hispanic populations. These racial differences reflect the average body weight for each racial group and further support the significant role that obesity and mechanical stress play in the aetiology of SCFE (4–7).

Patients with SCFE usually present with a limping and pain affecting the hip, groin, thigh, or knee, with limitation of internal rotation of the affected hip, obligatory external rotation of the hip in flexion and shortening of the leg; however, a vague presentation with mild, poorly localized pain in the lower limb can be also observed (1).

SCFE can be classified as unilateral or bilateral (33% of cases), acute (less than 3 weeks), chronic (symptoms for 3 weeks or more), or acute on chronic SCFE (in case of acute exacerbation of symptoms that have persisted for more than 3 weeks at first onset); SCFE can also be stable (90% of cases) or unstable, when the patient is unable to walk even with support (8–10).

Once the diagnosis of SCFE is made, the patient should avoid weight-bearing on the affected limb and be urgently referred to an orthopaedic surgeon. *in situ* fixation (ISF) is widely considered the standard of care for mild to moderate stable SCFE, although the optimal fixation method (single screw, double screw, or growing screw) remains debated. In contrast, the management of moderate to severe or unstable SCFE is still controversial. While ISF is still commonly performed, alternative strategies for acute SCFE, including serendipitous closed reduction followed by ISF, have also been described, theoretically reducing the morbidity associated with open procedures. Nevertheless, there is a growing trend towards the modified Dunn procedure (MDP), particularly in high-volume centres, as it allows anatomical realignment and may reduce the risk of femoroacetabular impingement, despite being technically demanding and not without complications (11–16).

Despite its considerable incidence in the pediatric population, a delayed diagnosis is not uncommon. Delayed recognition primarily increases the risk of further femoral epiphyseal slippage, which may necessitate more invasive and technically demanding procedures and is associated with a higher complication rate, ultimately compromising long-term prognosis (17, 18).

### Case presentation

A 16-year-old boy of African ethnicity was referred to our Rheumatology Department with a 6–month history of left hip and knee pain when walking. Initially, the pain was described as tolerable and associated with a mild limp, but in the previous month it had rapidly worsened with a complete refusal to walk. The clinical worsening followed an upper respiratory tract infection, without any trauma or atypical physical activity, and evolved over the course of a few days, with rapid onset of acute pain and inability to bear weight on the affected leg.

To investigate possible orthopaedic and neuropathic causes, he underwent an x-ray of the pelvis and lower limbs and a Magnetic Resonance Imaging (MRI) of the lumbosacral spine. Although the imaging studies were not available for direct review, the radiology reports indicated that the x-rays revealed no bone lesions and the MRI demonstrated no significant abnormalities, with all findings reported within normal limits. For the purposes of this report, it should be noted that the MRI was limited to the lumbosacral spine; therefore, the hips were not specifically evaluated or described. During this period, the patient exhibited a progressive decline in mood, characterized by reduced energy, persistent sadness, and diminished interest in previously enjoyable activities.

In the absence of detectable structural pathology and in light of the patient's deflected mood tone, functional etiologies were subsequently considered, prompting referral for paediatric neuropsychiatric evaluation. The patient was then discharged with a working diagnosis of functional pain.

A few days later, while at home, the patient developed swelling and stiffness of the left knee, associated with nocturnal pain and the need for assistance while walking. He subsequently underwent an external orthopaedic evaluation, which resulted in the temporary prescription of nonsteroidal anti-inflammatory drugs (NSAIDs) – Ibuprofen 5 mg/kg/day and Ketoprofen 1 mg/kg/day – and an elective MRI of the left knee. The treatment did not provide clinical benefit, and the patient was placed on a waiting list for the MRI. In the meantime, to further investigate the cause of knee pain, he was referred to our rheumatology centre, where he was hospitalized for additional diagnostic work-up.

Physical examination showed a complete functional limitation of both active and passive mobilization of the left hip and knee, with the left leg in neutral position. The patient was obese (weight 85 kg, >95th percentile; height 169 cm, 25–50th percentile; BMI 29.8 kg/m^2^, >97th percentile). Clinical presentation was consistent with mechanical pain, progressively worsening despite treatment with Naproxen (1 g/day) in the absence of fever. To further exclude rheumatological, onco-hematological and infectious causes, extensive investigations were performed, including complete blood count, electrolytes, inflammatory markers, metabolic panel, organ function tests, autoimmune profile, immunoglobulins and rheumatoid factor, yielding no conclusive findings. Consequently, imaging of the entire lower limb, rather than the knee alone, was undertaken.

Since the previous x-ray of the pelvis had been reported as negative, and to further exclude a possible neoplastic condition, a CT scan of the left lower limb was, prior to orthopaedic consultation, demonstrating posterior slippage of the femoral epiphysis.

The patient was then referred to the Orthopaedic Department for further management. Following orthopaedic evaluation, standard radiographs of the pelvis, including Lauenstein view, were obtained in the preoperative setting, confirming the diagnosis of slipped capital femoral epiphysis and demonstrating a severe slip. Radiographic findings are shown in Figure 1, while CT imaging is presented in Figure 2.

**Figure 1:**
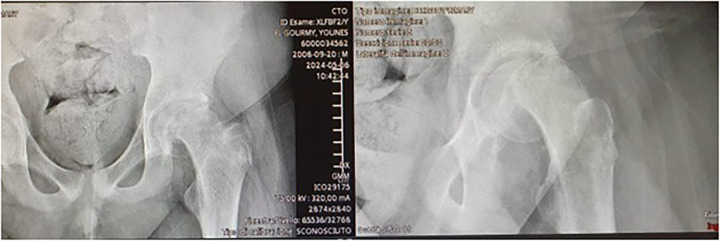
Preoperative radiographs obtained after orthopaedic evaluation.

**Figure 2:**
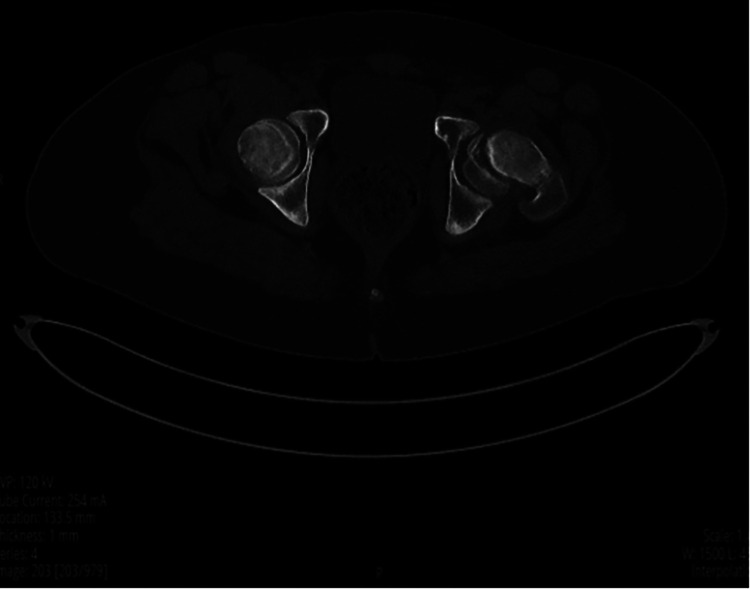
CT scan of the pelvis (bone window) highlighting the severity of the posterior slip and altered head–neck alignment.

Due to the severity of the clinical and radiographic condition, characterized by a marked posterior displacement of the femoral epiphysis and a Southwick angle in the severe range (>50°), the patient was not considered a suitable candidate for *in situ* fixation. In fact, ISF would not have allowed adequate correction of the deformity. Therefore, the patient underwent surgical treatment with a modified Dunn procedure (MDP) as described by Ganz et al. (19). This surgical technique involves a trigastric osteotomy of the greater trochanter and a surgical hip dislocation. The blood supply to the femoral head is preserved through two extended retinacular soft-tissue flaps.

Callus was removed to reduce tension over the medial femoral circumflex artery. After a careful subcapital realignment, the epiphysis was stabilized using a 3-mm threaded wire inserted anterogradely through the fovea capitis, along with two additional wires inserted in the opposite direction under fluoroscopic guidance. Intraoperative epiphyseal perfusion was assessed by drilling a 2-mm hole in the anterior portion of the femoral head both before and after epiphyseal reduction.

Postoperatively, the protocol included non-weight-bearing for three months, with restrictions on active abduction and passive adduction for 4–6 weeks (19). The patient underwent radiographic evaluations every 30 days for four months to monitor the positioning of the fixation devices and assess chondrolysis or avascular necrosis of the femoral head. At nine-months follow-up, no differences in the limb length were observed, the hip range of motion (ROM) was complete with physiological gait without limping and the x-ray showed no signs of avascular necrosis or chondrolysis of the hip joint or of femoroacetabular impingement (FAI). The patient underwent a rehabilitation physiotherapy program, which will last for at least one year.

As SCFE can be associated with endocrinopathies, a thorough assessment was performed. The physical examination showed a testicular volume of 6 mL, with stage PH2 pubic hair and absence of axillary hair. Considering the chronological age and the pubertal stage of the patient (Tanner G2 at 15 years and 7 months of age), gonadotropins and prolactin levels were also measured, showing normal results for age except for slightly reduced baseline testosterone; consequently, he underwent a gonadotropin-releasing hormone (GnRH) stimulation test that showed values compatible with the initial onset of pubertal development. After consultation with the endocrinologist, this was deemed to be consistent with delayed puberty. To monitor the progression of the pubertal development 6 months after the tests, a follow-up appointment was scheduled at the endocrinology department. During this visit, regular progression of pubertal development was observed, with an increase in testicular volume and growth of axillary and pubic hair, confirming the suspicion of constitutional delay in puberty with spontaneous resolution.

Lastly, given the condition of obesity, serial fasting blood glucose tests were also performed during the hospitalization, showing increased fasting glucose levels. Therefore, an oral glucose tolerance test was conducted showing signs of insulin resistance. The patient started a low-glycemic index diet and therapy with Metformin, and a specific follow-up plan was also established.

## Discussion

We report the case of a 16-year-old boy with SCFE, a condition comparable to a Salter–Harris type fracture involving the hypertrophic cellular zone of the physis. In SCFE, the growth plate is typically thickened, and chondrocytes across the proliferative, maturation, hypertrophic and degenerative zones are arranged in clusters rather than in orderly columns (20). Hormonal factors, particularly hypothyroidism and obesity, increase physeal susceptibility to shear stress, with an approximately four fold higher incidence in children with endocrinopathies (21).

This case is notable for multiple confounding factors contributing to delayed diagnosis. Initial mild knee pain during ambulation was underestimated by both the patient and the paediatrician, partly due to coexisting obesity and the common attribution of symptoms to functional joint overload. In such cases, weight loss is often recommended as first-line management. Furthermore, the concomitant upper respiratory tract infection raised the possibility of transient hip synovitis; however, this was ruled out given the patient's age. Mood depression, together with negative laboratory and imaging findings, further suggested a functional etiology. In addition, the patient was undergoing dual NSAID therapy prescribed by an external orthopaedic service, without significant clinical benefit and with potential risk of adverse effects including acute kidney injury and gastrointestinal irritation. Finally, the delayed diagnosis was partly influenced by the non-urgent nature of the orthopaedic imaging request, specifically the elective MRI of the knee and partial imaging of the lumbosacral spine, which contributed to the overall diagnostic delay. Physical examination showed the affected limb in a neutral position, rather than the typical external hip rotation, with global movement limitation and no clear asymmetry between the lower limbs; these findings were not consistent with the classic presentation of slipped capital femoral epiphysis. However, the patient's age, sex, ethnicity, obesity, and delayed pubertal development, in the context of an acute-on-chronic course, were important diagnostic clues. Earlier recognition might have enabled an earlier diagnosis, allowing less invasive treatment, fastest recovery, and reduced patient and family burden.

Diagnostic delay in chronic SCFE (often 3–12 months) may lead to progressive femoral head displacement, joint deformity and secondary coxarthrosis. Kocher et al. demonstrated a significant association between increasing diagnostic delay and greater slip angles (<30°: 10.0 weeks median; 30°–50°: 14.4 weeks median; >50°: 20.6 weeks median). Contributing factors to the delay include non-specific clinical presentation: patients with knee or distal thigh pain are more likely to be misdiagnosed, undergo unnecessary imaging, and present with more severe slips than those with localized hip pain (22). With regard to Loder's classification, 15 earlier diagnosis is more common in unstable SCFE, as weight-bearing inability and fracture-like symptoms prompt rapid evaluation and imaging. A similar pattern is observed in acute-on-chronic unstable SCFE, explaining the weaker correlation between delayed presentation and slip severity compared with chronic forms. Therefore, SCFE should be considered in all adolescents with poorly localized lower-limb pain, particularly in the presence of suggestive clinical history. Although CT imaging was performed earlier in our case, standard radiographs remain the first-line imaging modality for SCFE diagnosis. A key message is that, in paediatric patients presenting knee pain, the hip must always be examined and appropriately imaged with bilateral hip radiographs, including anteroposterior and frog-leg views (even bedside if ambulation is not possible).

The MDP provides superior deformity correction and good long-term outcomes compared with *in situ* fixation, with comparable osteonecrosis rates, restoring native anatomy, improving head–neck offset, and reducing the risk of anterior femoroacetabular impingement (cam-type) (19, 23). By limiting residual deformity and impingement, this approach may reduce the risk of early osteoarthritis and the potential need for premature total hip arthroplasty. Although outcomes of contemporary arthroplasty have improved, earlier joint replacement in young patients has historically been associated with increased revision risk, particularly due to aseptic loosening and polyethylene wear (24–29).

Finally, it is important to stress the association between SCFE and endocrinological dysfunctions. Therefore, a comprehensive evaluation is essential, addressing both the orthopaedic condition and the overall clinical context, with extension to endocrinological assessment based on the clinical findings.

## Conclusion

In summary, we described a 16-year-old boy with SCFE whose diagnosis was delayed due to multiple confounding factors, resulting in clinical deterioration. This case highlights that all adolescents presenting with hip, groin, or knee pain, whether acute or chronic, should be evaluated for SCFE, especially if overweight and in the absence of significant trauma, until definitively ruled out. Furthermore, comprehensive clinical assessment identified several associated endocrinopathies (insulin resistance and delayed puberty), underscoring the importance of a multidisciplinary approach to optimize management and long-term outcomes.

## Data Availability

The original contributions presented in the study are included in the article/Supplementary Material, further inquiries can be directed to the corresponding author.
